# Optimizing Aortic Valve Reoperations: Ministernotomy vs. Full Sternotomy

**DOI:** 10.3390/jcm14041213

**Published:** 2025-02-12

**Authors:** Elisa Mikus, Mariafrancesca Fiorentino, Diego Sangiorgi, Simone Calvi, Elena Tenti, Alberto Tripodi, Carlo Savini

**Affiliations:** 1 Cardiovascular Department, Maria Cecilia Hospital, GVM Care & Research, 48033 Cotignola, Italy; francescafiorentino@hotmail.it (M.F.); dsangiorgi@gvmnet.it (D.S.); scalvi@gvmnet.it (S.C.); etenti@gvmnet.it (E.T.); albertotripodi@hotmail.com (A.T.); csavini@gvmnet.it (C.S.); 2 Department of Experimental Diagnostic and Surgical Medicine (DIMEC), University of Bologna, 40126 Bologna, Italy

**Keywords:** minimally invasive surgery, aortic valve reoperation, unclamped patent internal mammary artery, reoperation

## Abstract

**Background**: The minimally invasive approach, performed via ministernotomy, is now often preferred for isolated aortic valve replacement (AVR). However, its benefits in patients with prior cardiac surgery remain unclear. This article compares traditional and minimally invasive surgery for isolated aortic valve replacement in reoperative cases. **Methods:** A retrospective analysis of 382 patients who underwent reoperative AVR between January 2010 and June 2024 divided them into two groups: 309 patients (80.1%) had a traditional full sternotomy, while 73 patients (19.1%) had minimally invasive AVR via upper ministernotomy. **Results:** Significant differences were noted between the groups. The full sternotomy group had a higher logistic EuroSCORE (SMD = 0.203), more patients with active endocarditis (SMD = 0.312), and a higher pacemaker rate. To minimize bias, inverse probability of treatment weighting (IPTW) was used. The minimally invasive group had shorter aortic cross-clamp (50 vs. 65 min, *p* < 0.001) and cardiopulmonary bypass times (62 vs. 85 min, *p* < 0.001), shorter intensive care unit (ICU) stays (*p* < 0.001), lower rates of acute renal failure (*p* = 0.001), and less blood loss (*p* < 0.001), but similar transfusion needs. Early mortality was higher in the full sternotomy group (4.5% vs. 1.6%, *p* = 0.025). **Conclusions:** Minimally invasive aortic valve reoperation via upper “J” sternotomy is as safe as full sternotomy. Patients experienced lower rates of acute renal failure and less postoperative bleeding, contributing to a safer recovery with decreased hospital mortality.

## 1. Introduction

Minimally invasive surgery (MIS) for aortic valve replacement (AVR) has increasingly become the standard approach in many specialized centers, consistently delivering excellent clinical outcomes. Among the minimally invasive techniques, the upper “J” ministernotomy, defined as a partial sternotomy typically extending from the manubrium to the third or fourth intercostal space, has demonstrated both safety and efficacy, as supported by numerous studies [1,2,3,4,5].

However, the potential advantages of MIS in the context of reoperative aortic valve replacement (redo-AVR) remain a subject of ongoing debate, particularly when patent coronary bypass grafts are present [6,7,8]. Reoperative isolated aortic valve replacement refers to patients undergoing isolated aortic valve replacement who have previously undergone cardiac surgery, including procedures involving the valves, mitral or aortic, coronary arteries, or other cardiac interventions.

Cardiac reoperations are generally associated with a higher perioperative risk of morbidity and mortality compared to primary surgeries. According to the existing literature, the reported hospital mortality rate for patients undergoing redo-AVR ranges from 0% to 6% [6,7,8,9,10]. The presence of patent bypass grafts, especially the left internal mammary artery (LIMA) graft, further exacerbates the risks, with sternal re-entry associated with a 2.5-fold increased risk of significant hemorrhage [11]. In these cases, minimally invasive approaches may offer an advantage by limiting the extent of surgical dissection and adhesion removal, potentially reducing the risk of graft injury and other complications.

When evaluating less invasive options for the treatment of isolated aortic valve disease in patients who have previously undergone cardiac surgery, it is essential to also consider, particularly in recent years, transcatheter aortic valve implantation (TAVI). TAVI offers reduced procedural trauma, making it an attractive option for patients, especially in cases of redo surgeries [12]. However, while TAVI procedures are feasible, they may be contraindicated in specific anatomical situations involving unfavorable positioning of the coronary ostia or complex configurations of previously implanted prosthetic valves [13]. Therefore, while TAVI is a promising alternative, it is crucial to carefully weigh its potential benefits and limitations in the context of each individual patient’s condition and surgical history. The decision on whether to proceed with a percutaneous or surgical approach is of critical importance and should be made by a heart team. Both options have distinct advantages and disadvantages, and the choice between them depends on various factors, including the patient’s overall health, the complexity of their previous surgeries, and their anatomical characteristics. The heart team’s collaborative decision-making ensures that the most appropriate and tailored treatment plan is chosen for each patient, optimizing outcomes while minimizing risks.

This study aims to compare the outcomes of conventional versus minimally invasive surgical approaches for redo-AVR, focusing on key indicators of morbidity and mortality. The analyzed postoperative complications include the incidence of acute renal failure, prolonged mechanical ventilation (>24 h), reintubation, deep sternal wound infections, sepsis, reoperation due to bleeding, and major cerebrovascular events. Additionally, metrics such as intensive care unit (ICU) length of stay, blood transfusion requirements, and overall hospital stay duration were evaluated to assess the overall impact on patient recovery and resource utilization.

## 2. Materials and Methods

The patients’ demographic details and primary baseline characteristics were collected and documented. These included variables such as age, gender, body mass index, creatinine clearance, preoperative status, cardiovascular risk factors, functional capacity, and left ventricular ejection fraction. Moreover, the EuroSCORE II was employed to estimate the risk of mortality linked to cardiac surgery. In addition to these baseline characteristics, intraoperative data and short-term postoperative outcomes were also documented.

### 2.1. Study Design and Outcomes

Between January 2010 and June 2024, a total of 3531 patients underwent isolated aortic valve replacement at our institution. Of these, 382 patients underwent isolated reoperative aortic valve procedures, which form the focus of this single-center, retrospective study. No formal calculation for sample size was performed; instead, all eligible patients within the specified time period were included in the analysis. The cohort was divided into two groups based on the surgical approach used. The first group consisted of 309 patients (80.1%) who underwent traditional full sternotomy, while the second group comprised 73 patients (19.1%), who underwent minimally invasive aortic valve replacement via an upper ministernotomy.

A comprehensive overview of the demographic and clinical characteristics of both groups is presented in Table 1. The study adhered to ethical guidelines, with approval obtained from the Romagna Ethics Committee on 12 December 2018 (protocol number 9507/2018). All participants provided written informed consent for the use of their data for clinical monitoring, quality control, and scientific research purposes. Data were collected from clinical records and systematically organized in a dedicated registry. Extensive measures were taken to reduce the occurrence of missing data. In cases where data were absent, this was attributed to gaps in the clinical documentation and was assumed to be missing completely at random. To ensure data integrity, only complete cases were included in the final analysis.

### 2.2. Surgical Technique

A minimally invasive approach consists of a partial upper resternotomy in a “J” shape performed at the third right intercostal space through a 6 cm skin incision. A transesophageal echocardiographic probe was placed in all patients for intraoperative assessment. Surgical dissection was primarily limited to the right side of the incision, with minimal dissection around the aorta and right atrium to facilitate cardiopulmonary bypass (CPB) cannulation and aortic cross-clamp placement. Continuous carbon dioxide (CO_2_) insufflation was utilized throughout the procedure. In patients undergoing aortic valve replacement (AVR) with a history of coronary artery bypass grafting (CABG) and a patent left internal mammary artery (LIMA) graft, no attempt was made to expose or exclude the LIMA, ensuring uninterrupted perfusion of the left side of the heart. Usually, a total central cannulation through the ascending aorta and the right atrium was performed. The left ventricle was routinely vented via the right upper pulmonary vein. Following aortic cross-clamping, antegrade normothermic blood cardioplegia was administered either into the ascending aorta or directly into the coronary ostia in cases of significant aortic insufficiency. The ascending aorta was incised transversely or obliquely, and the native or prosthetic valve was excised. The prosthetic valve was implanted using a standard technique, and the aortotomy was closed with a double-layer 4-0 suture. Ventricular pacing wires were positioned on the right ventricle, and the aortic cross-clamp was released. The patient was then gradually weaned from CPB, and intraoperative transesophageal echocardiographic was performed to confirm proper valve function. Cannulas were removed, and protamine was administered. The sternum was closed using steel wires, and subcutaneous sutures were applied for wound closure. In cases where a conventional full sternotomy was performed, the surgical technique remained comparable; however, LIMA graft isolation and clamping were rarely performed, and adhesion dissection was more extensive due to prior surgical interventions.

### 2.3. Statistical Analysis

After checking normal distribution with Shapiro–Wilk test, continuous variables were presented as medians with interquartile ranges (IQR) and compared using the Mann–Whitney test. Categorical variables were expressed as absolute numbers and frequencies, and comparisons were made using the chi-squared test or Fisher’s exact test, as appropriate. Multiple imputation by chained equations (MICE) was performed for variables with <50% of missing data; in particular, for missing data in categorical variables such as NYHA and CCS class a multinomial regression was used; binary covariates did not presented missing values.

To mitigate selection bias and ensure comparability between groups, the inverse probability of treatment weighting (IPTW) with covariate balancing propensity score (CBPS) method was applied; the following baseline characteristics were included in the model to balance the groups: age, gender, body mass index (BMI), hypertension, diabetes, dyslipidemia, smoking status, atrial fibrillation, pacemaker implantation, NYHA class, ejection fraction, endocarditis, stroke, transient ischemic attack (TIA), significant carotid artery disease, creatinine, chronic obstructive pulmonary disease (COPD), previous cardiac surgery, EuroSCORE, previous valve surgery, previous coronary artery bypass graft (CABG).

Absolute standardized mean differences (ASMD) were reported in order to assess balancing across groups; variables with ASMD < 0.2 were considered as balanced; variables not satisfying this criteria (pacemaker in the overall cohort) were included in all models to correct for potential unbalancing; weighted medians (IQR) and percentages were reported after IPW. Weighted logistic models were reported for binary outcomes, while weighted generalized linear models (GLMs) with appropriate families and link functions were used for continuous outcomes; weights were derived from IPTW; deviance residuals were analyzed for normality, while Cook’s distance was used to identify influential observations.

A sub-analysis considering only patients with previous CABG was also performed.

All analyses were performed using R version 4.4.0 (R Foundation for Statistical Computing, Vienna, Austria), with *p*-values < 0.05 considered as statistically significant.

## 3. Results

### 3.1. Study Population

The baseline characteristics of the entire patient population, stratified into two groups based on the surgical approach, are summarized in Table 1. Significant differences were observed between the groups. Specifically, patients who underwent a standard full sternotomy had a higher logistic EuroSCORE (standardized mean difference (SMD) = 0.203), a greater proportion of patients with active endocarditis (SMD = 0.312), and a higher percentage of those with a permanent pacemaker (SMD = 0.272). Conversely, the same group exhibited a lower incidence of chronic obstructive pulmonary disease, which was statistically significantly different from the second group (SMD = 0.215) (Table 1).

**Table 1:** Patient baseline characteristics.

	Full Sternotomy	Minimally Invasive	*p*	SMD
n	309	73		
Age, median [IQR]	72 [66, 77]	74 [64, 78]	0.472	0.049
Male, n (%)	197 (63.8)	52 (71.2)	0.275	0.160
Body mass index, median [IQR]	25.9 [23.8, 28.9]	26.9 [24.5, 29.6]	0.148	0.175
Hypertension, n (%)	240 (77.7)	60 (82.2)	0.433	0.113
Diabetes, n (%)	63 (20.4)	14 (19.2)	0.873	0.030
dyslipidemia, n (%)	184 (59.5)	45 (61.6)	0.791	0.043
Preoperative atrial fibrillation, n (%)	38 (12.3)	14 (19.2)	0.131	0.190
Pacemaker, n (%)	11 (3.6)	0 (0.0)	0.134	0.272
Ejection fraction %, median (IQR)	55 [50, 61]	55 [50, 65]	0.902	0.008
Active endocarditis, n (%)	101 (32.7)	14 (19.2)	0.024	0.312
Stroke, n (%)	16 (5.2)	3 (4.1)	1.000	0.051
Previous TIA, n (%)	7 (2.3)	2 (2.7)	0.684	0.030
Significant carotid artery disease, n (%)	1 (0.3)	1 (1.4)	0.346	0.114
Creatinine, median [IQR]	1.00 [0.85, 1.20]	1.02 [0.88, 1.20]	0.814	0.096
Chronic obstructive pulmonary disease, n (%)	32 (10.4)	13 (17.8)	0.104	0.215
Logistics EuroSCORE, median (IQR)	16.63 [9.13, 30.78]	15.64 [8.63, 25.19]	0.291	0.203
Previous valvuar surgery, n (%)	264 (85.4)	49 (67.1)	0.001	0.441
Previous bypass surgery, n (%)	54 (17.5)	23 (31.5)	0.010	0.331

IQR: interquartile range; TIA: transient ischemic attack.

Additionally, there were notable differences in the type of previous cardiac surgery between the two groups. Patients in the first group, who underwent a median sternotomy, had a significantly lower percentage of previous coronary artery bypass grafting (CABG) with patent grafts (SMD = 0.331). On the other hand, this group showed a significantly higher proportion of patients who had previously undergone valvular surgery (SMD = 0.441). Characteristics of the two groups after inverse probability of treatment weighting (IPTW) are reported in Table 2.

### 3.2. Postoperative Outcomes and Mortality

The surgical technique employed for the minimally invasive approach has been previously documented in the literature [14]. All postoperative outcomes are detailed in Table 3.

The analysis comparing the two groups, following the application of inverse probability of treatment weighting (IPTW), revealed statistically significant differences across several key variables. When comparing the outcomes of patients who underwent median sternotomy to those who received minimally invasive surgery, notable differences were observed in procedural times. Specifically, the aortic cross-clamp time was significantly shorter in the minimally invasive group, averaging 50 min compared to 65 min in the median sternotomy group (*p* < 0.001). Similarly, the duration of cardiopulmonary bypass was also reduced in the minimally invasive cohort, with an average time of 62 min versus 85 min for the sternotomy group (*p* < 0.001). Additionally, the length of stay in the intensive care unit (ICU) was shorter for patients undergoing minimally invasive surgery, with a *p*-value of 0.015. Further advantages in outcomes for patients undergoing ministernotomy were noted in terms of postoperative complications. The incidence of acute renal failure was lower in the minimally invasive group (*p* = 0.029), as was the need for renal replacement therapy, including dialysis or continuous veno-venous hemofiltration (CVVH) (*p* = 0.002). Moreover, there were reduced postoperative blood losses in this group (*p* < 0.001), although the number of packed red blood cell transfusions was similar across both groups. Conversely, no statistically significant differences were observed in other parameters, such as the occurrence of acute myocardial infarction or the onset of atrial fibrillation.

Regarding early mortality (in-hospital or within 30 days), there was a higher rate observed in patients treated with median sternotomy (4.5%) compared to those undergoing the minimally invasive approach (1.6%). However, this difference did not reach statistical significance (*p* = 0.097).

**Figure 1:**
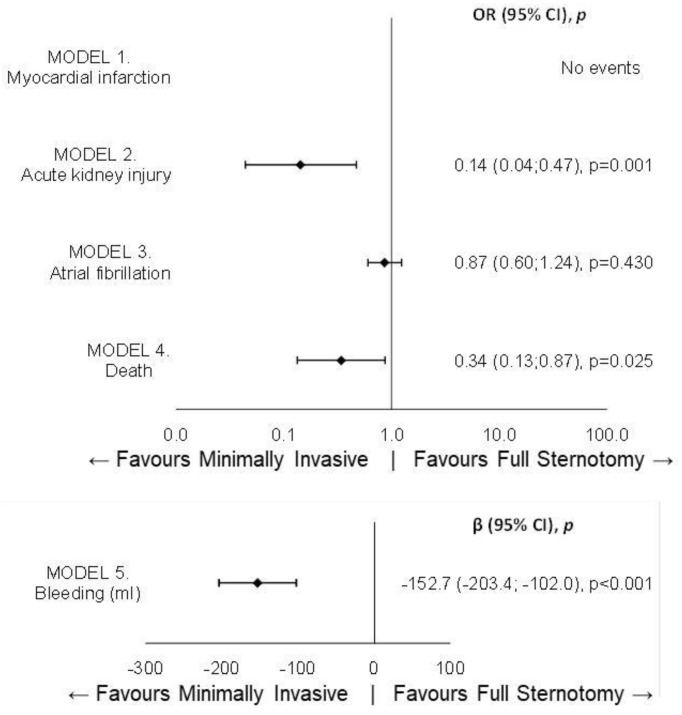
Forest plot for in-hospital outcomes.

After performing the multivariable analysis, as shown in Figure 1, the minimally invasive approach proved to be protective both against renal failure (OR = 0.143, 0.043, 0.471, *p* = 0.001) and bleeding (β = −152.7, −203.4, −102.0, *p* < 0.001), as well as hospital mortality (OR = 0.341, 0.133, 0.872, *p* = 0.025).

**Figure 2:**
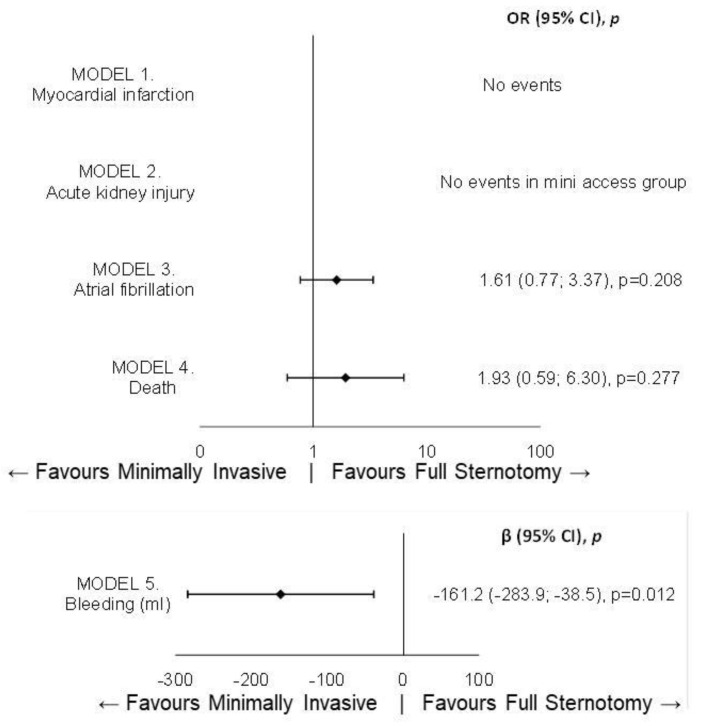
Forest plot for in-hospital outcomes in the previously CABG subgroup.

We then analyzed the subgroup of patients who had previously undergone coronary artery bypass grafting with patent grafts, in order to determine whether the minimally invasive approach was protective against mortality or perioperative myocardial infarction. From the multivariable analysis, neither outcome was found to be influenced by the type of surgical approach, with the limitation that this subgroup was extremely small (54 patients with the traditional approach vs. 23 treated with the minimally invasive approach) (Figure 2). It was confirmed only that the minimally invasive approach was associated with lower postoperative blood loss (β =−161.2, −283.9; −38.5, *p* = 0.012).

## 4. Discussion

Reoperative cardiac surgery carries inherently increased operative risks, primarily due to the potential for damage to cardiac structures. This can result in complications such as bleeding, the need for transfusions, and prolonged operative times, particularly in patients with prior coronary artery bypass surgery involving patent grafts. In this context, minimally invasive aortic valve replacement presents a promising alternative to the traditional full sternotomy, aiming to reduce surgical trauma and facilitate quicker recovery.

One of the key advantages of minimally invasive techniques in reoperations, particularly in patients with patent left internal mammary artery (LIMA) grafts, is the reduced need for extensive dissection, which lowers the risk of graft injury and myocardial infarction (12). Traditional methods often require temporary occlusion of the LIMA graft or the use of alternative strategies such as deep hypothermia, but these approaches come with significant risks, including inadequate myocardial protection [15,16,17,18]. In contrast, miAVR performed with unclamped grafts under normothermic conditions has demonstrated excellent outcomes, with reduced rates of myocardial infarction and shorter aortic cross-clamp times, as confirmed by our findings [8,14,19].

The technical benefits of a minimally invasive approach are particularly evident in patients with patent grafts, but this method is also advantageous in those with prior valve replacements. A smaller incision simplifies both the opening and closure of the sternotomy, significantly reducing the risk of sternal instability and infection. The upper ministernotomy approach provides optimal exposure of the aortic root, and in reoperative settings, it minimizes the extent of pericardial dissection. The dissection is typically confined to the ascending aorta and a small portion of the right atrium for cannulation, which helps reduce the risk of injury to the right ventricle. Central cannulation can be employed effectively even with the minimally invasive approach, and it is not contraindicated. Despite the smaller operative field, the procedure still allows for adequate exposure of the aortic valve, as evidenced by the low extracorporeal circulation times observed in our series. Thus, the advantages of minimally invasive surgery can be achieved without compromising the effectiveness of the procedure.

Our results also confirm the safety of minimally invasive approaches in high-risk populations, including patients with endocarditis. Contrary to concerns, endocarditis, even when complicated by aortic abscesses, does not represent a contraindication to minimally invasive surgery, further broadening the applicability of this approach.

When evaluating less invasive options like transcatheter aortic valve implantation (TAVI), several key factors must be taken into account. TAVI provides rapid symptom relief with less procedural trauma, making it an appealing choice for high- or intermediate-risk patients, particularly in cases of redo surgeries [12]. However, it comes with its own set of risks, including paravalvular leaks and potentially limited long-term durability compared to surgically implanted valves [19]. In particular, valve-in-valve TAVI procedures, though feasible, may be contraindicated in certain anatomical scenarios, such as unfavorable positioning of the coronary ostia or complex configurations of previously implanted prosthetic valves [13].

While TAVI excels in providing faster recovery and is often the preferred treatment for patients for whom open surgery poses excessive risk, surgical aortic valve replacement also continues to offer several advantages in the redo field. These include longer follow-up periods, excellent long-term outcomes, and low mortality rates, which range between 0% and 6%, according to various studies [6,7,8,9,10,20,21,22]. Additionally, a recent study published by Raschpichler M. et al. highlighted that although redo surgery for aortic valve deterioration is associated with higher rates of early mortality and stroke in univariate analysis, much of this increased risk can be attributed to comorbidities. Therefore, they conclude that patients undergoing redo aortic valve replacement have outcomes similar to those undergoing primary SAVR [22].

Moreover, it is important to recognize that TAVI is not without limitations. In certain patients, valve-in-valve TAVI procedures may be more risky or even contraindicated due to specific anatomical factors, such as the structure of the native valve, the configuration of the previously implanted prosthesis, or the proximity of the coronary ostia. These challenges highlight the continued relevance of surgical approaches, particularly in cases where anatomy or previous interventions make TAVI less suitable. Therefore, careful, patient-specific evaluation should guide the choice between surgical and percutaneous techniques, weighing the benefits of each approach according to the patient’s unique anatomy, surgical history, and overall health.

### Limitations

This study has several limitations. Firstly, it was non-randomized, and the data were collected retrospectively. The choice of surgical approach was primarily determined by the discretion of the surgeon. Naturally, this introduces a potential source of bias; however, it is within the common constraints of studies focused on surgical techniques. Such studies often reflect the surgeon’s personal preference in selecting the approach they are most comfortable with or believe to be the most effective. Additionally, this study is based on the experience of a single surgical center and lacks a formal sample size calculation, which may limit the generalizability and statistical power of the findings.

## 5. Conclusions

In conclusion, despite the extensive literature on the use of minimally invasive surgery for aortic valve procedures and its advantages, there are very limited data available regarding the use of ministernotomy in the case of reoperations. The results of our study demonstrate that minimally invasive aortic valve surgery can be performed safely with excellent outcomes, even in reoperative settings. The mortality rate for minimally invasive approaches (1.6%) compares favorably with that of full sternotomy (4.5%), offering a viable, if not superior, alternative to traditional surgery. This approach reduces unnecessary surgical trauma, enhances recovery through reduced ventilation time, and minimizes the postoperative bleeding. Importantly, the absence of postoperative myocardial infarctions in our study confirms that leaving patent LIMA and/or RIMA grafts unclamped is a safe strategy. Minimally invasive surgery thus represents an effective, less invasive option for patients requiring aortic valve reoperation, offering both short- and long-term benefits without compromising surgical efficacy.

## Figures and Tables

**Table 2:** Patient baseline characteristics after inverse probability weighting.

	Full Sternotomy	Minimally Invasive	*p*	SMD
n	388.38	371.37		
Age, median [IQR]	71 [65, 77]	73 [64, 78]	0.591	0.020
Male, n (%)	255.0 (65.7)	243.0 (65.4)	0.973	0.005
Body mass index, median [IQR]	26.0 [23.8, 29.1]	26.06 [23.8, 28.5]	0.788	0.005
Hypertension, n (%)	301.1 (77.5)	288.1 (77.6)	0.995	0.001
Diabetes, n (%)	75.6 (19.5)	69.6 (18.7)	0.901	0.018
Dyslipidemia, n (%)	233.4 (60.1)	223.4 (60.2)	0.995	0.001
Preoperative atrial fibrillation, n (%)	52.9 (13.6)	48.8 (13.2)	0.919	0.013
Pacemaker, n (%)	11.0 (2.8)	0.0 (0.0)	0.002	0.241
Ejection fraction %, median [IQR]	55 [50, 60]	55 [50, 60]	0.993	0.004
Active endocarditis, n (%)	110.2 (28.4)	99.1 (26.7)	0.810	0.038
Stroke, n (%)	18.8 (4.8)	16.8 (4.5)	0.924	0.015
Previous TIA, n (%)	8.9 (2.3)	6.9 (1.9)	0.805	0.030
Significant carotid artery disease, n (%)	1.1 (0.3)	1.1 (0.3)	0.975	0.002
Creatinine, median [IQR]	1.00 [0.86, 1.20]	1.00 [0.77, 1.13]	0.442	0.012
Chronic obstructive pulmonary disease, n (%)	43.3 (11.1)	41.2 (11.1)	0.993	0.001
Logistics EuroSCORE, median [IQR]	15.54 [8.46, 26.12]	16.03 [10.17, 26.54]	0.806	0.042
Previous valvuar surgery, n (%)	314.6 (81.0)	299.5 (80.7)	0.948	0.009
Previous bypass surgery, n (%)	81.5 (21.0)	79.5 (21.4)	0.940	0.010

IQR: interquartile range; TIA: transient ischemic attack.

**Table 3:** Surgical data and postoperative outcomes.

	Full Sternotomy	Minimally Invasive	*p*	OR
n	388.38	371.37		(95% CI), *p*
CPB time, median [IQR]	85 [65, 114]	62 [53, 80]	<0.001	/
Cross clamp time, median [IQR]	65 [51, 84]	50 [40, 67]	<0.001	/
Mechanical valve, n (%)	132.2 (33.9)	124.6 (33.4)	0.952	/
Biological valve, n (%)	256.2 (66.1)	247.2 (66.6)	0.952	/
Biological sutureless valve, n (%)	42.7 (10.9)	47.8 (12.9)	0.673	/
Blood transfusions, n (%)	254.4 (65.5)	243.4 (65.5)	0.997	1.011 (0.748; 1.366), 0.946
Acute myocardial infarction (%)	0.0 (0.0)	0.0 (0.0)	1.000	Not assessable
Cardiac tamponade, n (%)	3.1 (0.8)	0.0 (0.0)	0.093	Not assessable
Stroke, n (%)	6.0 (1.5)	9.5 (2.6)	0.512	1.623 (0.578; 4.561), 0.358
TIA, n (%)	1.0 (0.3)	3.0 (0.8)	0.396	Not assessable
Bleeding requiring chest reopening, n (%)	12.0 (3.1)	13.3 (3.6)	0.850	1.235 (0.549; 2.781), 0.610
Acute kidney injury, n (%)	22.3 (5.7)	3.2 (0.8)	0.029	0.143 (0.043; 0.471), 0.001
Dialysis, n (%)	12.7 (3.3)	0.0 (0.0)	0.002	Not assessable
Permanent pmk implantation, n (%)	27.0 (7.0)	28.5 (7.7)	0.837	1.121 (0.646; 1.947), 0.684
New onset atrial fibrillation, n (%)	80.3 (20.7)	69.5 (18.7)	0.728	0.865 (0.604; 1.240), 0.430
Ventilation > 48 h, n (%)	29.3 (8.1)	17.4 (5.5)	0.518	0.664 (0.358; 1.321), 0.194
Gut ischemia, n (%)	3.7 (1.0)	0.0 (0.0)	0.099	Not assessable
Wound complications, n (%)	8.3 (2.1)	4.4 (1.2)	0.583	0.616 (0.187; 2.024), 0.425
Sepsis, n (%)	2.3 (0.6)	15.1 (4.1)	0.032	6.754 (1.694; 26.928), 0.007
Ventilation time (hours), median [IQR]	10 [6, 16]	7 [5, 14]	0.041	−0.40 (−1.12; 0.16), 0.180
Postoperative bleeding (mL), median [IQR]	400 [250, 550]	266 [154, 400]	<0.001	−152.7 (−203.4; −102.0), <0.001
Hospital mortality, n (%)	17.3 (4.5)	6.0 (1.6)	0.097	0.341 (0.133; 0.872), 0.025
ICU (days), median [IQR]	2.00 [1.92, 3.29]	2.00 [1.58, 2.91]	0.015	−0.20 (−0.28; −0.12), <0.001
Hospital stay (days), median [IQR]	7.0 [7.0, 10.0]	7.7 [7.0, 10.0]	0.865	0.12 (0.08; 0.17), <0.001

CPB: cardiopulmonary bypass; mL: milliliters; ICU: intensive care unit; IQR: interquartile range; TIA: transient ischemic attack.

## Data Availability

The data presented in this study are available on request from the corresponding author. The data are not publicly available due to data protection directive 95/46/EC.

## Abbreviations

The following abbreviations are used in this manuscript:

ASMD	absolute standardized mean differences
AVR	aortic valve replacement
BMI	body mass index
CABG	coronary artery bypass graft
CO_2_	carbon dioxide
CPB	cardiopulmonary bypass
CBPS	covariate balancing propensity score
COPD	chronic obstructive pulmonary disease
CVVH	continuous veno-venous hemofiltration
GLMs	generalized linear models
ICU	intensive care unit
IPTW	inverse probability of treatment weighting
IQR	interquartile ranges
LIMA	left internal mammary artery
MIS	minimally invasive surgery
MICE	multiple imputation by chained equations
redo-AVR	reoperative aortic valve replacement
RIMA	right internal mammary artery
SMD	standardized mean difference
TAVI	transcatheter aortic valve implantation
TIA	transient ischemic attack

## References

[1] Khalid S., Hassan M., Ali A., Anwar F., Siddiqui M.S., Shrestha S. (2024). Minimally invasive approaches versus conventional sternotomy for aortic valve replacement in patients with aortic valve disease: A systematic review and meta-analysis of 17,269 patients. Ann. Med. Surg..

[2] El-Andari R., Fialka N.M., Shan S., White A., Manikala V.K., Wang S. (2024). Aortic Valve Replacement: Is Minimally Invasive Really Better? A Contemporary Systematic Review and Meta-Analysis. Cardiol. Rev..

[3] Woldendorp K., Doyle M.P., Bannon P.G., Misfeld M., Yan T.D., Santarpino G., Berretta P., Di Eusanio M., Meuris B., Cerillo A.G. (2020). Aortic valve replacement using stented or sutureless/rapid deployment prosthesis via either full-sternotomy or a minimally invasive approach: A network meta-analysis. Ann. Cardiothorac. Surg..

[4] Murtuza B., Pepper J.R., Stanbridge R.D., Jones C., Rao C., Darzi A., Athanasiou T. (2008). Minimal access aortic valve replacement: Is it worth it. Ann. Thorac. Surg..

[5] Brown M.L., McKellar S.H., Sundt T.M., Schaff H.V. (2009). Ministernotomy versus conventional sternotomy for aortic valve replacement: A systematic review and meta-analysis. J. Thorac. Cardiovasc. Surg..

[6] Santarpino G., Berretta P., Kappert U., Teoh K., Mignosa C., Meuris B., Villa E., Albertini A., Carrel T.P., Misfeld M. (2020). Minimally Invasive Redo Aortic Valve Replacement: Results from a Multicentric Registry (SURD-IR). Ann. Thorac. Surg..

[7] Orlov O.I., Kaleda V.I., Shah V.N., Nguyen C., Orlov C.P., Sicouri S., Takebe M., Goldman S.M., Plestis K.A. (2019). Ministernotomy aortic valve surgery in patients with prior patent mammary artery grafts after coronary artery bypass grafting. Eur. J. Cardio-Thorac. Surg..

[8] Gaeta R., Lentini S., Raffa G., Pellegrini C., Zattera G., Viganò M. (2010). Aortic valve replacement by ministernotomy in redo patients with previous left internal mammary artery patent grafts. Ann. Thorac. Cardiovasc. Surg..

[9] Paupério G.S., Pinto C.S., Antunes P.E., Antunes M. (2012). Aortic valve surgery in patients who had undergone surgical myocardial revascularization previously. Eur. J. Cardio-Thorac. Surg..

[10] Dobrilovic N., Fingleton J.G., Maslow A., Machan J., Feng W., Casey P., Sellke F.W., Singh A.K. (2012). Midterm outcomes of patients undergoing aortic valve replacement after previous coronary artery bypass grafting. Eur. J. Cardio-Thoracic Surg..

[11] Follis F.M., Pett S.B., Miller K.B., Wong R.S., Temes R.T., Wernly J.A. (1999). Catastrophic hemorrhage on sternal reentry: Still a dreaded complication?. Ann. Thorac. Surg..

[12] Nasir M.M., Ikram A., Usman M., Sarwar J., Ahmed J., Hamza M., Farhan S.A., Siddiqi R., Qadar L.T., Shah S.R. (2024). Valve-in-Valve Transcatheter Aortic Valve Replacement Versus Redo-Surgical Aortic Valve Replacement in Patients with Aortic Stenosis: A Systematic Review and Meta-analysis. Am. J. Cardiol..

[13] Tran J.H., Itagaki S., Zeng Q., Leon M.B., O’Gara P.T., Mack M.J., Gillinov A.M., El-Hamamsy I., Tang G.H.L., Mikami T. (2024). Transcatheter or Surgical Replacement for Failed Bioprosthetic Aortic Valves. JAMA Cardiol..

[14] Mikus E., Calvi S., Tripodi A., Dozza L., Lamarra M., Del Giglio M. (2015). Minimally invasive reoperative aortic valve replacement. Ann. Cardiothorac. Surg..

[15] Gillinov A.M., Casselman F.P., Lytle B.W., Blackstone E.H., Parsons E.M., Loop F.D., Cosgrove D.M. (1999). Injury to a patent left internal thoracic artery graft at coronary reoperation. Ann. Thorac. Surg..

[16] Byrne J.G., Aranki S.F., Couper G.S., Adams D.H., Allred E.N., Cohn L.H. (1999). Reoperative aortic valve replacement: Partial upper hemisternotomy versus conventional full sternotomy. J. Thorac. Cardiovasc. Surg..

[17] Savitt M.A., Singh T., Agrawal S., Choudhary A., Chaugle H., Ahmed A. (2002). A simple technique for aortic valve replacement in patients with a patent left internal mammary artery bypass graft. Ann. Thorac. Surg..

[18] Battellini R., Rastan A.J., Fabricius A., Moscoso-Luduena M., Lachmann N., Mohr F.W. (2007). Beating heart aortic valve replacement after previous coronary artery bypass surgery with a patent internal mammary artery graft. Ann. Thorac. Surg..

[19] Smith R.L., Ellman P.I., Thompson P.W., Girotti M.E., Mettler B.A., Ailawadi G., Peeler B.B., Kern J.A., Kron I.L. (2009). Do you need to clamp a patent left internal thoracic artery left anterior descending graft in reoperative cardiac surgery?. Ann. Thorac. Surg..

[20] Schnackenburg P., Saha S., Ali A., Horke K.M., Buech J., Mueller C.S., Sadoni S., Orban M., Kaiser R., Doldi P.M. (2024). Failure of Surgical Aortic Valve Prostheses: An Analysis of Heart Team Decisions and Postoperative Outcomes. J. Clin. Med..

[21] Khaladj N., Shrestha M., Peterss S., Kutschka I., Strueber M., Hoy L., Haverich A., Hagl C. (2009). Isolated surgical aortic valve replacement after previous coronary artery bypass grafting with patent grafts: Is this old fashioned technique obsolete?. Eur. J. Cardiothorac. Surg..

[22] Raschpichler M., Kiefer P., Otto W., Noack T., Gerber M., De Waha S., Dashkevich A., Leontyev S., Misfeld M., Borger M.A. (2024). Redo surgical aortic valve replacement for bioprosthetic structural valve deterioration. Eur. J. Cardio-Thorac. Surg..

